# Inconsistent Protective Efficacy and Marked Polymorphism Limits the Value of *Schistosoma japonicum* Tetraspanin-2 as a Vaccine Target

**DOI:** 10.1371/journal.pntd.0001166

**Published:** 2011-05-31

**Authors:** Wenbao Zhang, Jun Li, Mary Duke, Malcolm K. Jones, Ling Kuang, Jianfeng Zhang, David Blair, Yuesheng Li, Donald P. McManus

**Affiliations:** 1 Molecular Parasitology Laboratory, Australian Centre for International and Tropical Health and Nutrition, Queensland Institute of Medical Research, Brisbane, Queensland, Australia; 2 Parasite Cell Biology Laboratory, Queensland Institute of Medical Research, Brisbane, Queensland, Australia; 3 The School of Veterinary Science, The University of Queensland, St Lucia, Queensland, Australia; 4 School of Marine and Tropical Biology, James Cook University, Townsville, Queensland, Australia; 5 Hunan Institute of Parasitic Diseases, Yueyang, China; National Institutes of Health, United States of America

## Abstract

**Background:**

*Schistosoma mansoni* tetraspanin 2 (Sm-TSP-2) has been shown to be strongly recognized by IgG1 and IgG3 antibodies from individuals putatively resistant to schistosome infection, but not chronically infected people, and to induce high levels of protection against challenge infection in the murine model of schistosomiasis. Amplification by PCR of homologous sequences from male and female *S. japonicum* worms showed the presence of 7 different clusters or subclasses of *S. japonicum* TSP-2. We determined the protective efficacy of one subclass – Sj-TSP-2e.

**Methodology/Principal Findings:**

Following the alignment of 211 cDNAs, we identified 7 clusters encoding *S. japonicum* TSP-2 (*Sj-TSP-2*) based on sequence variation in the large extracellular loop (LEL) region with differing frequency of transcription in male and female worms. Quantitative PCR analysis revealed elevated expression of *Sj-TSP-2* in adult worms compared with other life cycle stages. We expressed in *E. coli* the LEL region of one of the clusters which exhibited a high frequency of transcription in female worms, and showed the purified recombinant protein (Sj-TSP-2e) was recognised by 43.1% of sera obtained from confirmed schistosomiasis japonica patients. Vaccination of mice with the recombinant protein induced high levels of IgG1 and IgG2 antibodies, but no consistent protective efficacy against challenge infection was elicited in three independent trials.

**Conclusions/Significance:**

The highly polymorphic nature of the *Sj-TSP-2* gene at the transcriptional level may limit the value of Sj-TSP-2 as a target for future *S. japonicum* vaccine development.

## Author Summary

*Schistosoma mansoni* tetraspanin 2 (Sm-TSP-2) is considered a lead target for vaccine development against schistosomiasis mansoni because: (1) It is located in the schistosome tegument and is involved in tegument formation; (2) It is strongly recognized by IgG1 and IgG3 antibodies from individuals putatively resistant to schistosome infection, but not chronically infected people, and (3) It induces high levels of protection against challenge infection in the mouse model. We amplified 211 homologous TSP-2 sequences from male and female *S. japonicum* worms, which revealed 7 different cDNA subclasses. We expressed in *E. coli* a region of one of the clusters which exhibited a high frequency of transcription in female worms, and showed the purified recombinant protein (Sj-TSP-2e) was recognised by 43.1% of sera obtained from confirmed schistosomiasis japonica patients. Vaccination of mice with the recombinant protein induced high levels of IgG1 and IgG2 antibodies, but no consistent protective efficacy against challenge infection was elicited in three independent trials. The highly polymorphic nature of the *Sj-TSP-2* gene at the transcriptional level may limit the value of Sj-TSP-2 as a target for future *S. japonicum* vaccine development. Further analysis of the distribution of the different subclasses/alleles of the *Sj-TSP-2* gene in *S. japonicum* populations from different endemic areas would be informative.

## Introduction

There is now growing agreement that integrated control, which could include the use of an effective vaccine combined with chemotherapy and other measures, is the optimum direction that the future control of schistosomiasis should follow [1], [2]. Vaccine development against schistosomiasis has been guided by the fact that irradiated cercariae confer >80% protection in experimental animal models and natural hosts including mice, rats, rabbits, sheep and bovines [3].

A number of promising anti-schistosome vaccine candidates exist but they may prove not to be the most effective and it is, therefore, important to continue to identify new target antigens and to explore alternative vaccination strategies to improve vaccine efficacy [2]. A reporter-based signal sequence capture technique identified two *S. mansoni* tetraspanins (Sm-TSP-1 and TSP-2) [4], both proteins being expressed in the tegument membrane [5]. The large extracellular loop (LEL) of Sm-TSP-2, in particular, provided high levels of protection as a recombinant vaccine in the mouse model of schistosomiasis, and both proteins were strongly recognized by IgG1 and IgG3 from putatively resistant individuals but not chronically infected people [5]. A subsequent study showed that Sm-TSP-2 plays a role in the formation of the *S. mansoni* tegument [6], which is critically important for the parasite's survival [7].

Following these studies on Sm-TSP-2, genes and gene subclasses encoding TSP-2 homologues were isolated from *S. japonicum* (*Sj-TSP-2*) [8]. However, mice vaccinated with the same LEL of Sj-TSP-2 present in Sm-TSP-2 were not protected following a challenge infection with *S. japonicum* [8]. The study showed also that *Sj-TSP-2* is highly polymorphic and, as a result, these authors argued against further development of Sj-TSP-2 as a vaccine candidate against schistosomiasis japonica [8]. Subsequently, however, another group used a similar *Sj-TSP-2* sequence to produce recombinant Sj-TSP-2 and obtained significant (46–58% efficacy) in mice vaccinated with the protein and then challenged [9]. In light of these contradictory results, we cloned and sequenced a slightly different *Sj-TSP-2* sequence (*Sj-TSP-2e*), and expressed and purified its recombinant product. The Sj-TSP-2e protein was recognised by sera from some patients positive for *S. japonicum* infection, but the molecule did not protect mice using either a high (35 cercariae) or low (12 cercariae) dose of *S. japonicum* challenge infection.

## Materials and Methods

### Ethics statement

The conducts and procedures involving animal experiments were approved by the Animal Ethics Committee of the Queensland Institute of Medical Research. Ethical approval for using human sera for this study was granted by the Ethics Committee of Hunan Institute of Parasitic Diseases, Hunan, China.

### Parasites

*Oncomelania hupensis* infected with *S. japonicum* were obtained from an endemic area in Anhui Province, China. Adult worms were collected from two rabbits (each experimentally infected with 200 cercariae) 7 weeks post-infection. Cercariae and schistosomula were collected as previously described [10]. The worms were stored in RNAlater (Ambion, Foster City, CA) at −70°C until use.

### Total RNA extraction and cDNA synthesis

Total RNA was extracted from either a pool of 20 males or 30 female worms using an RNAeasy Mini Kit (Qiagen, Hilden, Germany) to remove potential DNA contamination. The kit was also used to extract total RNA from cercariae and schistosomula. First strand cDNA was synthesized using a Sensiscript RT Kit (Qiagen).

### Cloning and sequence alignment of *S. japonicum-TSP-2*

Homology searches using BLAST showed that *S. japonicum* has a range of sequences homologous to *S. mansoni TSP-2*. The cDNA sequences are highly conserved at both termini. Accordingly, we designed two primers: upstream; 5′-ATGGCTCTCGGGTGTGGATACAAG-3′ and downstream; 5′-CTATTCATCATCGCCTCGTTTTATAGCC-3′ to amplify the ORF of cDNA using the first strand cDNA from males and females, respectively, as templates. The PCR products were separated by running an agarose gel and a DNA band matching the designated size was cut and extracted using a Qiaquick Gel Extraction kit (Qiagen). The DNA was then ligated into a cloning pGEMT vector (Promega, Madison, WI). We sequenced 100 clones from each of the male and female cDNA preparations to determine the distribution of homologous sequences between male and female *S. japonicum* worms. We used ClustalW2 (http://www.ebi.ac.uk/Tools/clustalw2/index.html) and Bioedit (http://www.mbio.ncsu.edu/bioedit/bioedit.html) for sequence alignment.

### Phylogenetic analysis

Inferred amino acid sequences of the LEL portions of the sequenced clones were aligned with homologues (TSP-2) identified by BLAST searches in GenBank and with sequences reported by Cai et al [8]. The sole relevant sequence from *S. mansoni* was used as the outgroup. The alignment of the LEL region consisted of 77 sites of which 37 were variable. Phylogenetic analysis was done using MrBayes [11]. The Jones model for amino-acid substitution+G (gamma distribution of rates with four rate categories)+I (proportion of invariant sites) was used. Values for I and for the shape parameter (alpha) for the gamma distribution were estimated from the data during the runs. Two million generations (2 runs each of 4 chains) were specified and the chain was sampled every 1000 generations. The first 10% of sampled trees were discarded as burnin. The standard deviation of split frequencies was well below 0.01 by the end of the analysis.

### Stage expression of *S. japonicum-TSP-2* by quantitative PCR

We used quantitative PCR to determine the expression level of *Sj-TSP-2* in four life cycle stages (adult male, adult female, cercaria and schistosomulum) of *S. japonicum* using the up-stream primer: 5′-ACAATGTTGTGGTGCCGAATCGCC-3′ and down-stream primer: 5′- CTATTCATCATCGCCTCGTTTTATAGCC-3′. After first strand cDNA synthesis, all the cDNA samples were diluted to a concentration of 10 ng/µl. Subsequently, 5 µl aliquots were combined with 10 µl of SYBR Green, 3 µl of water and 2 µl (5 pmol) of the forward and reverse primers. Each experiment was performed in triplicate. Expression profiles of *Sj-TSP-2* in the different stages were obtained by real time PCR using a Rotor Gene (6000) real time PCR machine (Qiagen) and data were analysed by Rotor Gene 6 Software. *S. japonicum* NADH-ubiquinone reductase was employed as a house keeping gene [10] in the quantitative analysis using the primers: 5′-CGAGGACCTAACAGCAGAGG -3′ and 5′- TCCGAACGAACTTTGAATCC-3′.

### Recombinant protein expression

To express the large extracellular loop (LEL) sequence (Glu107 to His180) of Sj-TSP-2e, we designed a pair of primers (5′-GAAAAGCCGAAGGTGAAAAGACA-3′ and 5′-GCGGTGCTTTTTAGTCAGATCGGTGA-3′) to amplify the target fragment and subcloned the sequence into the pBAD/Thio-TOPO plasmid (Invitrogen). The fragment was fused in-frame with the N-terminal thioredoxin (Thi) and the C-terminal V5 epitope (V5) and 6His tags. The plasmid was transformed into *E. coli*. Subsequent protein expression under native conditions was conducted as recommended by the manufacturer. The recombinant fusion protein (Sj-TSP-2e-Thi) from *E. coli* lysates was purified under non-denaturing conditions using 6His affinity chromatography (BD Biosciences). The identities of the purified proteins were confirmed by Western blotting with antibodies to thioredoxin and 6His epitopes. *S. mansoni* TSP-2 was kindly provided by Professor Alex Loukas and was expressed as described [5]. We designed a pair of primers: 5′-GGGAATTCAAAATGTCTGAAAAGCCGAAGGTGAAAAGACA -3′ and 5′-GCCTCGAGGTGCTTTTTAGTCAGATCGGTGAC-3′ to subclone the LEL fragment into the pPICZ-c vector (Invitrogen) and transformed into *Pichia* yeast (Invitrogen) according to the manufacturer's instructions. The generated protein was fused with a His tag at the C terminal for affinity purification.

### Immunolocalization

Murine anti-Sj-TSP-2e serum was produced as previously described [12]. The serum was absorbed with laboratory made “beads” binding a bacterial lysate having the Thi protein tag to remove antibodies against the Thi and His tag proteins and bacterial proteins as described [12]. We then used Western blotting to confirm that the anti-Sj-TSP-2e serum did not recognize either the Thi tag or proteins in the bacterial lysate. For immunolocalization, we collected fresh adult *S. japonicum* worms from rabbits. The worms were fixed in 100% methanol, embedded in Tissue-Tek Optimal Cutting Temperature (OCT) compound (ProSciTech), and cryostatically sectioned into 7.0-µm sections. The sections were blocked with 5% (w/v) skimmed milk powder in PBS containing 0.1% (v/v) Tween 20 (PBST) as a blocking solution (SMPT), incubated firstly with mouse anti–Sj-TSP-2 serum diluted 1∶25 with SMPT, and then by rabbit antibody to mouse IgG conjugated to Cy2 (Jackson ImmunoResearch; diluted 1∶150 in SMPT). Sections were counterstained with DAPI (Sigma; 0.1 µg/ml in PBS), which stains nuclei. We mounted the slides with DAKO mounting medium and examined them using a confocal microscope (Leica TCS SP2). Normal mouse serum and murine serum raised against thioredoxin tagged with 6 His were used as controls.

### SDS-PAGE and Western blot analysis

A soluble adult worm antigen preparation (SWAP) of *S. japonicum* was prepared from worms collected from rabbits after 5 washes with PBS. The worms were resuspended in PBS containing protease inhibitor cocktail (Sigma, St. Louis, MO), disrupted on ice using a homogenizer, and sonicated five times using 10-second bursts. The suspension was centrifuged at 60,000 *g* at 4°C for 1 h, and the supernatant was stored at −80°C until use. The pellet was washed twice with PBS and dissolved in 1% SDS (w/v) in PBS and heated at 56°C for 30 min. The supernatant was used as a source of insoluble parasite proteins after centrifugation at 60,000 g and at room temperature for 1 h.

For Western blotting, the protein preparations, including SWAP, insoluble parasite proteins and recombinant proteins were run on SDS-PAGE. The separated proteins were then transferred onto a nitrocellulose membrane. After blocking in SMPT, the membrane was incubated at 37°C for 1 h with the murine anti-Sj-TSP-2 serum, diluted 1 in 1000, then incubated with HRP labelled rabbit antibodies against mouse IgG for 1 h after 3 washes with PBST. 4-chloro-1-naphthol was used as substrate to develop the colour reaction using protocols previously described [13], [14].

### Screening human and mouse sera by ELISA

Nunc Maxisorp Surface 96-well plates were coated with 100 µl of 10 µg/ml of whole parasite protein preparation (5 µg/ml of SWAP and 5 µg/ml of insoluble proteins for screening human sera), 2 µg/ml of Sj-TSP-2 or Thi tag (for screening human and mouse sera) in 0.06 M NaCO_3_, pH 9.6 overnight at 4°C. The plates were then blocked with SMPT at 37°C for 1 h after two washes with PBS.

Human sera from confirmed schistosomiasis japonica cases (n = 72) by microscopy with fecal egg identification and serology against adult worm antigens before treatment [15] were collected from Han Chinese patients from endemic areas of Hunan province, People's Republic of China (PRC) and 24 normal sera were collected from healthy Han Chinese from Xinjiang, PRC, where schistosomiasis is not endemic. The human sera were diluted 1 in 100 with SMPT. The remaining ELISA processing steps were essentially as previously described [13], [14].

Mouse serum isotypes were measured by ELISA based on methods previously described with mini modification [16], [17]. In brief, mouse sera were diluted to 1 in 500 with in PBST for detecting IgA, IgG and IgM antibodies, and 1∶50 for IgE. 100 µl of each diluted serum was added to each well, and plates were incubated at 37°C for 1 h for IgA, IgG and IgM detection or overnight at 4°C for IgE. This was followed by 4 washes with PBST. Then anti-mouse IgG or IgG subclass was added at 1∶3000 dilution, IgE at 1∶2500 dilution and IgM at 1∶10000 dilution (Invitrogen) and the plates incubated for 1 h at 37°C, except for IgE detection when the plates were incubated overnight at 4°C. The plates were then washed five times with PBST. The assays were developed in 2,2-azino-di-(ethyl-benzithiozolin sulphonate) (ABTS) (Sigma) substrate solution for 30 min. The optical density (OD) of the colour that developed in the plates was read at 405 nm using an ELISA reader (VersaMax, East Falmouth, MA).

### Mouse vaccination trials

Each vaccine trial undertaken comprised three groups of mice, including two experimental groups each vaccinated separately with Sj-TSP-2e and Sm-TSP-2 and one control group vaccinated with thioredoxin (Thi) tag protein. Each group comprised 10 female CBA/J mice (18–22 g in weight and 6–8 weeks old). Recombinant Sj-TSP-2e and Sm-TSP2 (25 µg per dose in 25 µl) were formulated with an equal volume of either Freunds complete (primary) or Freunds incomplete (two boosts at two weekly intervals) adjuvants and the preparations were subcutaneously injected into the mice. All mice were anaesthetised with a mixture of ketamine (100 mg/kg body weight) and xylazine (20 mg/kg body weight) 2 weeks after the final vaccine injection and challenged percutaneously with *S. japonicum* cercariae by the cover slip method. The mice were challenged with 35 (high dose) or 12 (low dose) cercariae.

The mice were sacrificed using CO_2_ at 7 weeks post-challenge and necropsied to determine worm and liver egg burdens. Mice were perfused with PBS to remove worms from the mesenteric veins as described [18] and the numbers of male and female adult parasites present counted. The mouse livers were weighed and digested with 10 ml 5% (w/v) potassium hydroxide overnight at 37°C on a rocking platform. Liver eggs were then counted in aliquots by light microscopy. Fecal egg counts were determined by collecting fecal samples from each of the mice over a 48-h period of 4 collections (0.3–0.5 g/mouse) before perfusion. The fecal samples from each of the mice were pooled, weighed and mixed by vortexing in PBS and then rotating overnight at 4°C. Fecal pellets were obtained by centrifugation at 500 *g* for 10 min, resuspended in 50 ml PBS and filtered through 600-µm and then 250-µm mesh sieves. The filtered material was pelleted by centrifugation at 500 *g* for 10 min and resuspended in 10 ml PBS. A 100-µl aliquot was pipetted onto a microscope slide and the number of eggs in each aliquot counted. For each sample, 10 aliquots (1000-µl of egg suspension) were counted to obtain the total number of eggs in 10 ml and then the number of eggs-per-gram (e.p.g.) for each of the mice determined.

The level of protection was expressed as a percentage based on the reduction in worm burden and liver and faecal eggs in the groups vaccinated with the Sj-TSP-2e and Sm-TSP-2 proteins compared with the control groups.

### Statistical analysis

A nonparametric Mann-Whitney *U*-test was used for analysis of the vaccine trial data because of the relatively small sample size in each experiment. Spearman's rank correlation was used to analyze the correlation between worm burden/egg numbers and serum optical-density values in ELISA. We compared the median values of each vaccine test group with the control groups to calculate reductions in worm burden, and fecal and liver eggs. P<0.05 was taken to indicate statistically significant differences.

## Results

### A region in the large extracellular loop region of Sj-TSP-2 is variable

**Figure 1:**
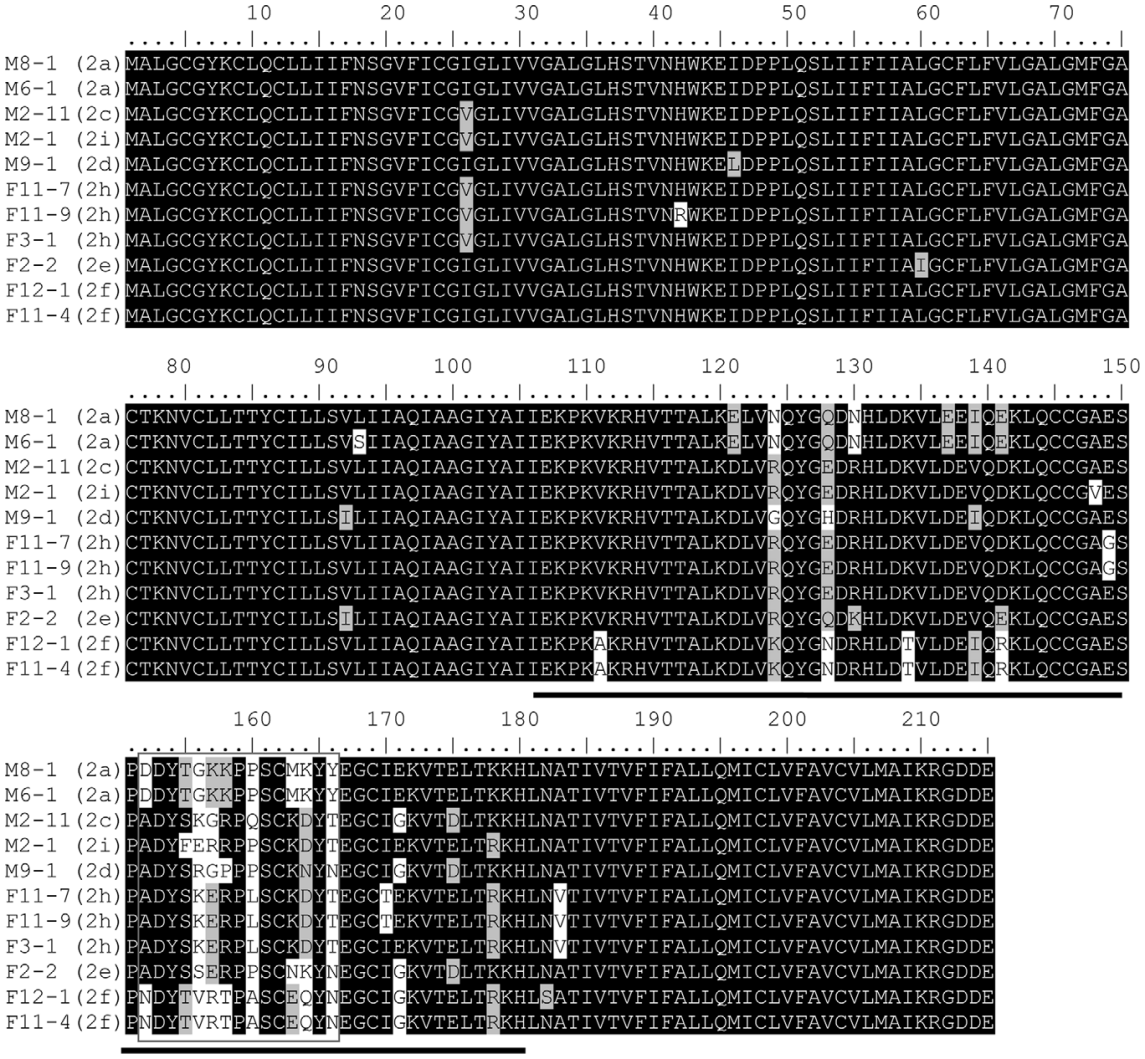
Protein sequence alignment of *Schistosoma japonicum* tetraspanin 2 (Sj-TSP-2) isolated from adult worms. The variable region is boxed. The large extracellular loop region is indicated by a solid line. Sj-TSP-2a (M8-1 and M6-1, GenBank accession numbers JF264973 and JF264974), Sj-TSP-2c (M2-11, JF264975), Sj-TSP-2d (M9-1, JF264977), Sj-TSP-2e (F2-2, JF264978), Sj-TSP-2f (F12-1, JF264982; F11-4, JF264983), Sj-TSP-2h (F11-7, JF264979; F11-9, JF264980; F3-1, JF264981) and Sj-TSP-2i (M2-1, JF264976) were from individual male (M) and female (F) worms. For instance, M8-1 represents number 8 male worm and number 1 clone.

BLAST analysis showed that a number of *S. japonicum* sequences deposited in the GenBank and other databases were homologous to *Sm-TSP-2*. The Sj-TSP-2 sequences at both the N and C terminal ends are nearly identical, which allowed us to design primers to amplify the full-length ORF of the cDNAs. We sequenced 108 clones from male and 103 from female adult worms (Table 1). The ORFs of the sequences were identical in size, encoding a precursor protein of 215 amino acids. Protein sequence analysis showed that all the proteins have a similar structure containing four transmembrane domains and two loops (Figures S1 and S2). Whereas the N and C termini are highly conserved, the large loop region, which was used in the Sm-TSP-2 vaccine studies by Tran et al [5], is highly variable. The variation results in 7 clusters and Fig. 1 and Table 1 show representatives of each of the clusters. We compared the sequences with a previous study [8] and showed that 5 of the clusters were identical to those earlier reported as subclasses Sj-TSP-2a, c, d, e and f (Table 1). We identified two additional clusters, Sj-TSP-2h and Sj-TSP-2i (Table 1), but were unable to amplify any of the Sj-TSP-2b or Sj-TSP-2g sequences described previously [8].

**Table 1:** Subclass distribution of *S. japonicum* tetraspanin 2 (Sj-TSP-2) in male and female worms.

Sj-TSP-2	Variable region*	Clones in			
		Males	(%)	Females	(%)
Sj-TSP-2a	TGKK	16	(14.8)	0	(0)
Sj-TSP-2b	TGEK	0	(0)	0	(0)
Sj-TSP-2c	SKGR	27	(25)	9	(8.7)
Sj-TSP-2d	SRGP	32	(29.6)	36	(34.9)
Sj-TSP-2e	SSER	4	(3.7)	24	(23.3)
Sj-TSP-2f	TVRT	13	(12)	11	(10.7)
Sj-TSP-2g	TVRT‡	0	(0)	0	(0)
Sj-TSP-2h†	SKER	12	(11.1)	8	(7.8)
Sj-TSP-2i†	FERR	4	(3.7)	15	(14.6)
Total	-	108	-	103	-

*, The variable region of the Sj-TSP-2 protein sequence from amino acid 155 to 158 of the full-length sequences.

**†:** , Two previously undescribed sequences;

**‡:** , same as Sj-TSP-2f in the variable region, which Cai et al. [8] suggested was a different subclass as it contains substitutions in other regions.

The gene clusters were differentially transcribed in male and female *S. japonicum*; Table 1 shows their frequency of transcription in male and female worms. *Sj-TSP-2a* was specifically expressed in males whereas *Sj-TSP-2e* was more prominent in female worms (Table 1).

**Figure 2:**
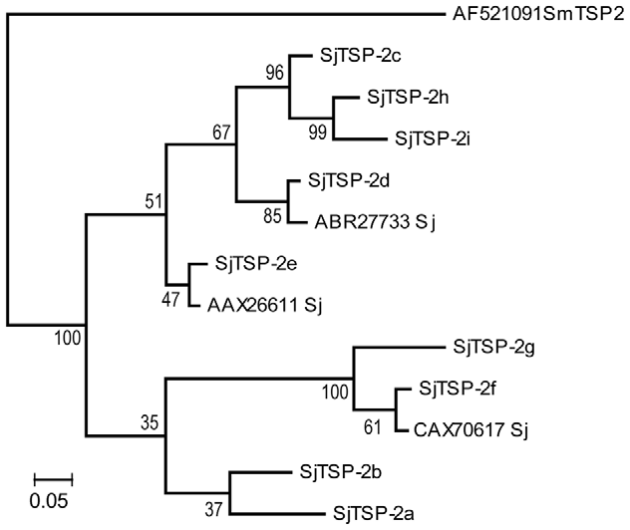
Phylogenetic analysis of Sj-TSP-2 clusters with homologues. Protein sequences corresponding to the LEL region of Sj-TSP-2 encoded by 9 clusters of Sj-TSP-2 cDNAs were aligned with additional homologues found by BLAST searches in GenBank and others reported by Cai et al [8]. Analysis was done as stated in the text. Clade credibility (posterior probability) values are shown at nodes. Protein sequence data reported in this paper are available in the GenBank, EMBL and DDBJ databases under the accession numbers ABR27733, AAX26611 and CAX70617.

A phylogenetic tree of the Sj-TSP-2 sequences found using MrBayes is shown in Figure 2. Twelve different sequences for the LEL were found in *S. japonicum*. These differed from each other by between 1 and 22 sites. Pair-wise differences between any sequence from *S. japonicum* and the homologue from *S. mansoni* ranged from 28 to 33.

**Figure 3:**
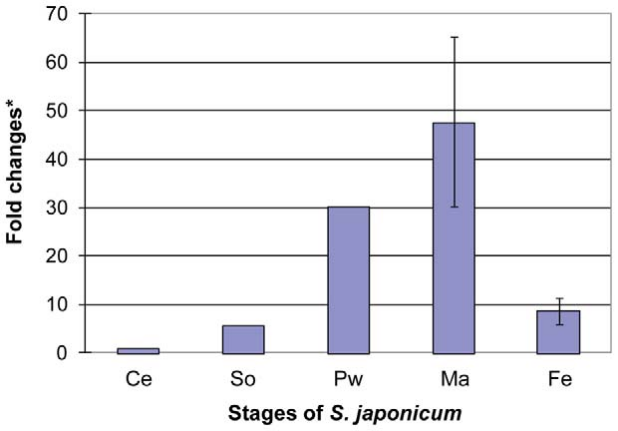
Expression levels of Sj-TSP-2e by real-time PCR. cDNAs were amplified with mRNA isolated from different stages of *S. japonicum* using specific primers designed from the conserved regions of Sj-TSP-2e. Ce, cercariae; So, schistosomula; Pw, paired adult worms; Ma, males; Fe, females. The bars (and *, X axis) show the fold changes compared with the cercarial stage. We used NADH-ubiquinone reductase as a house-keeping gene to calculate the number of copies of the gene expressed in each of the stages, and then converted these to fold changes by comparison with the number of copies in cercariae.

We used real time PCR to quantify the expression levels of *Sj-TSP-2* in different stages of *S. japonicum* with a pair of primers designed from the identical regions of the clusters. The analysis showed that *Sj-TSP-2* was differentially transcribed in different stages of the parasite, with the gene being expressed 6 and 30 times higher in schistosomula and adult worms, respectively, than in cercariae. In addition, expression of *Sj-TSP-2* was 47-fold higher in adult males and 8.7 times higher in adult females compared with the cercariae (Fig. 3).

### Expression and purification of Sj-TSP-2e

**Figure 4:**
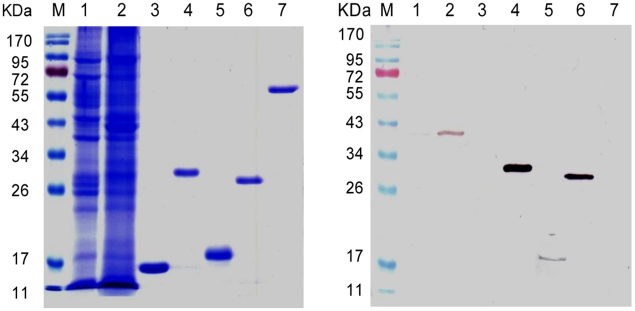
Purification and recognition of recombinant Sj-TSP-2e. Left panel, SDS-PAGE of soluble and insoluble proteins extracted from adult *S. japonicum* and recombinant proteins. Right panel: Recognition of recombinant Sj-TSP-2e and native proteins by antibodies in hyper immune mouse serum raised against recombinant Sj-TSP-2e. Lane M, protein markers; Lanes 1 and 2, soluble and insoluble proteins of *S. japonicum*; lane 3, Thi; lane 4, Sj-TSP-2e-Thi; lane 5, Sj-TSP-2e expressed in *Pichia* yeast; lane 6, Sm-TSP-2e-Thi; lane 7, Sj-23-GST-His as a control protein.

As the LEL region of Sm-TSP-2 was used successfully as a vaccine for *S. mansoni* [5], we expressed the homologous region of Sj-TSP-2e using the pBAD/Thio-TOPO plasmid expression system; this expresses the target protein fused with thioredoxin. As described earlier, we cloned several clusters of Sj-TSP-2 but we expressed the Sj-TSP-2e subclass due to its high frequency of transcription in females (Table 1), indicating its role in female development, which is a target for vaccine development [2]. The purity of Sj-TSP-2e and Sm-TSP-2 are shown in Fig. 4. In an effort to increase antigenicity, we also employed a yeast expression system to express the LEL region of Sj-TSP-2e (Fig. 4).

### Antibody recognition of Sj-TSP-2e

We used three methods to characterize *Sj-TSP-2e* expression in *S. japonicum*. Firstly, we extracted the whole repertoire of parasite proteins including soluble and insoluble proteins. We then used Western blotting to probe the native protein with hyper immune mouse serum prepared against recombinant Sj-TSP-2e (Fig. 4). A band of 38 kDa was recognized in the native protein preparations (from worms collected from rabbits) by the immune serum; a strong band was evident in the insoluble protein preparation and a weaker band in the soluble fraction (Fig. 4).

**Figure 5:**
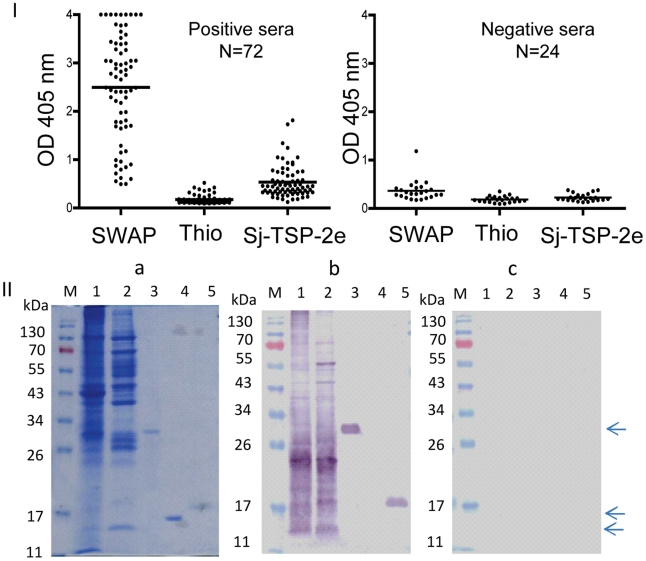
Recognition of recombinant Sj-TSP-2e by human sera. I: ELISA IgG screen of 72 sera collected from positive schistosomiasis japonica patients and 24 normal human sera (negative controls) probed by protein preparation from *S. japonicum* adult worms (SWAP), recombinant tag protein thioredoxin (Thio) and Sj-TSP-2e. II. Panel a: SDS-PAGE of soluble and insoluble protein extracted from adult *S. japonicum* and recombinant Sj-TSP-2e. Panels b and c: Western blot analysis with pooled sera randomly selected from confirmed schistosomiasis japonica patients (n = 15) and pooled sera from negative control subjects (n = 15) from northern China. Lane M, protein markers; lanes 1 and lane 2, soluble and insoluble proteins from adult *S. japonicum*; lane 3, Sj-TSP-2e (arrowed); lane 4, thioredoxin tag (Thi) (arrowed); lane 5, Sj-TSP-2e expressed in *Pichia* yeast (arrowed).

Secondly, recombinant Sj-TSP-2e LEL was recognized by a serum pool, from 15 randomly selected patients, with confirmed *S. japonicum* infection, but the pool did not bind the Thi tag protein (Fig. 5I). We coated the recombinant protein and native proteins onto ELISA plates for screening 72 individual sera collected from positive schistosomiasis japonica patients. All the sera recognised the native proteins with different OD values (Fig. 5II). We reacted the sera with Sj-TSP-2e-Thi protein; 43.1% (31/72) of the sera had OD values 2.5-fold higher than the average OD values of negative sera (Fig. 5II) and only one of the 72 sera reacted positively with the Thi tag protein alone.

**Figure 6:**
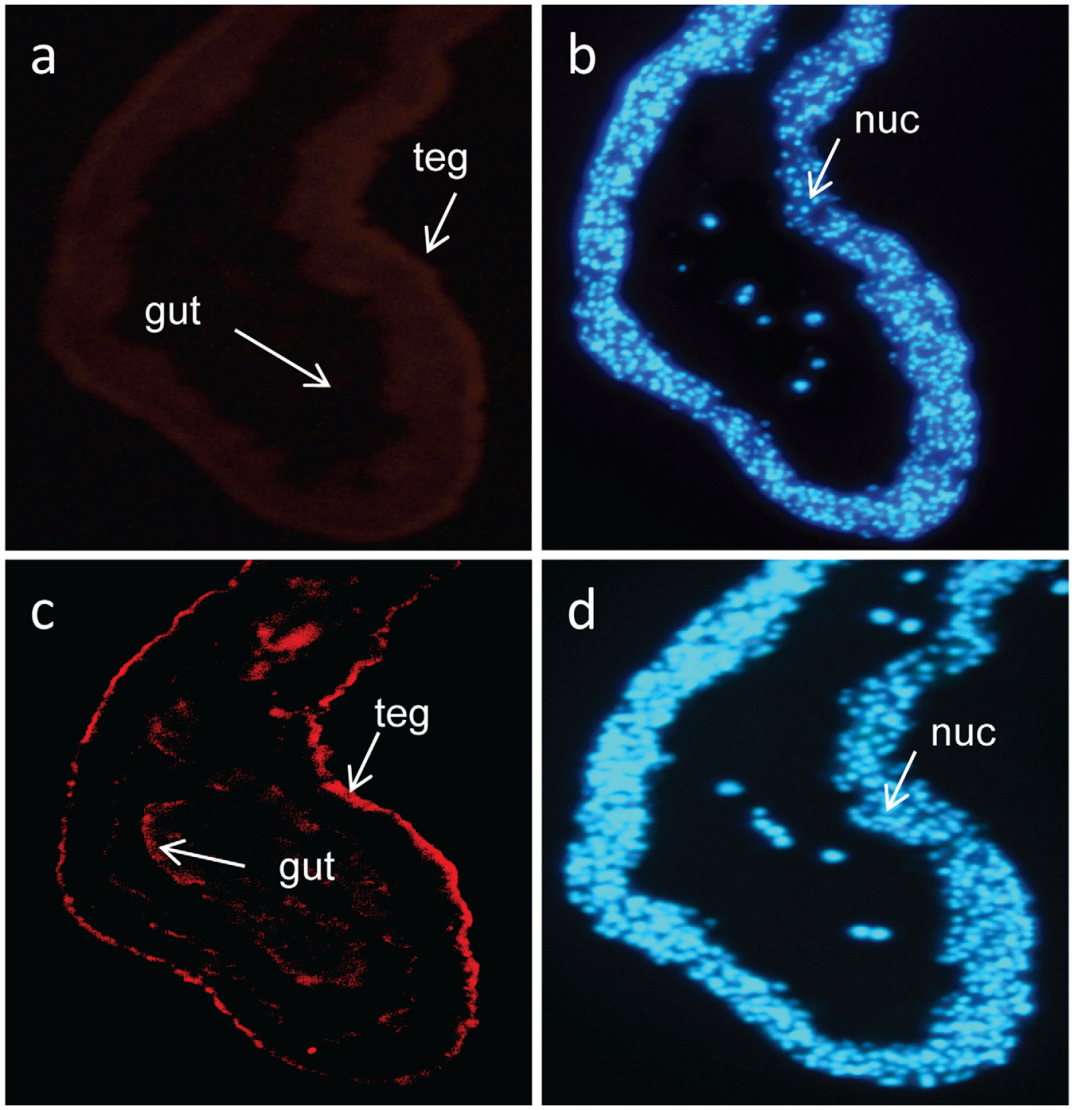
Immunolocalization of *S. japonicum* Sj-TSP-2e in adult female worms. Parasite sections were reacted with specific antibodies produced in mice against recombinant Sj-TSP-2e. The antibodies that specifically bound to the sections were probed with an anti-mouse IgG labelled with Cy3 conjugate (a, c). Red fluorescence in panel c indicates Sj-TSP-2e is located in the parasite tegument (teg); anti-thioredoxin (fusion protein tagged with 6His) antibodies in panel a did not react. DAPI to label nuclei (nuc in blue) (b, d) was used as a quality control marker for the sections. Tegument, teg.

Thirdly, we used the hyper-immune mouse serum prepared against recombinant Sj-TSP-2e to localize Sj-TSP-2e in female *S. japonicum*. The results showed that specific antibodies against Sj-TSP-2e bound to the tegument and gut of adult female worms (Fig. 6). Sections of male worms showed minimal staining (data not shown).

### Vaccination of mice with Sj-TSP-2e

**Figure 7:**
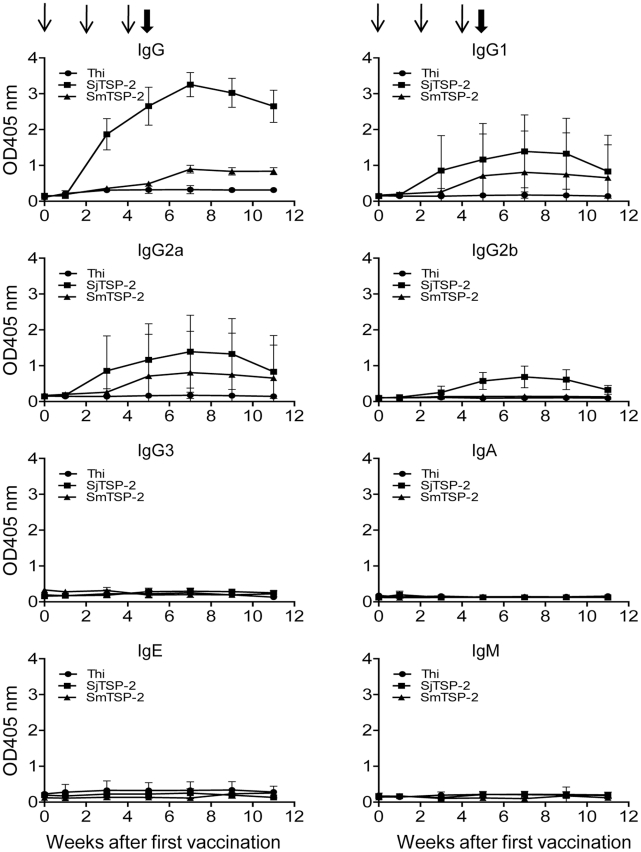
Antibody isotype levels in mice challenged with 35 *S. japonicum* cercariae. Mouse serum anti-Sj-TSP-2e (fused with thioredoxin, Thi) antibodies were determined by ELISA after primary vaccination with *S. japonicum*-TSP-2e (Sj-TSP-2e) or *S. mansoni*-TSP-2 (Sm-TSP-2). Thioredoxin was used as control protein in the vaccine trials. The thin arrows indicate vaccination time points and the bold arrows indicate the time point of cercarial challenge.

In order to determine whether Sj-TSP-2e had any protective efficacy, three trials were undertaken; these comprised two experiments where mice were challenged with a high dose of 35 cercariae, and one experiment where mice were challenged with a low dose of 12 cercariae. Murine serum samples collected throughout the course of the vaccine trials with Sj-TSP-2e did not react in ELISA with thioredoxin fused with 6His alone (data not shown), indicating the very poor antigenicity of this tag protein. In contrast, the vaccinated mice generated high levels of IgG against SjT-SP-2e after the second injection and these peaked after the third injection; the serum titres dropped slightly just prior to perfusion of the mice (Fig. 7). Of the IgG subclasses, IgG1 antibodies were dominant and IgG2a antibodies were also increased significantly (Fig. 7). IgA, IgE and IgM were at background levels throughout the three trials (Fig. 7). IgG antibodies, including IgG1 and IgG2, in the sera collected from mice vaccinated with Sm-TSP-2 recognized Sj-TSP-2e (Fig. 7), indicating that the two antigens may share similar epitopes.

Table 2 shows the vaccination results of the three independent trials. In the first trial, Sj-TSP-2e induced a low but significant reduction in worm burden (36.4%, P<0.001) and liver eggs (26.5%, P<0.05). Accordingly, we repeated the experiment but could not corroborate the results in the second trial. We considered that the challenge with 35 cercariae was so high that damage to the established immune system in the mice could have occurred, and thus we used a low dose challenge with 12 cercariae. Compared with the control, however, no significant protection (Table 2) was induced by Sj-TSP-2e in the vaccinated mice; there were 54.4% and 68.9% reductions in liver and fecal eggs, respectively, in the vaccinated mice but the reductions were not significant when compared with the control groups. Sm-TSP-2 induced 70.9% reduction in liver egg numbers but this was also not significant (P>0.05) compared to the control group. In addition, antibody levels (before challenge and perfusion) showed no correlation with worm burdens or egg counts (data not shown). Overall, the trials showed that Sj-TSP-2e did not induce any consistent protection in mice against a *S. japonicum* challenge infection.

**Table 2:** Vaccination results of mice vaccinated with SjTSP-2e against challenge infection with *Schistosoma japonicum* cercariae.

Trial		Worm burden				Eggs in whole liver				Eggs in one gram of faeces			
Protein	Range (worm pairs)	Mean ± SD	Med	Redu (%)	P value	Mean ± SD	Med	Redu (%)	P value	Mean ± SD	Med	Redu (%)	P value
**Trial 1 (HDC)**													
Thi	18–29(7.8)	23.2±3.1	22	**-**	**-**	53488±16407	59508	**-**	**-**	ND		**-**	**-**
Sj-TSP2	3–14(3.9)	15.4±3.2	14	**36.4**	**0.0004**	37868±16286	43764	**26.5**	**0.019**	ND		**-**	**-**
Sm-TSP2	4–18(4.9)	19.1±4.4	19.5	**11.4**	**0.032**	40041±32551	28743	**51.7**	**0.075**	ND		**-**	**-**
**Trial II (HDC)**													
Thi	10–32(10.1)	20.0±6.3	21	**-**	**-**	97407±36537	112216	**-**	**-**	4791±2969	5714	**-**	**-**
Sj-TSP2	2–23(6.8)	14.3±6.0	16.5	**21.4**	**0.065**	95833±31066	99339	**11.5**	**0.842**	4509±2870	4067	**28.8**	**0.806**
Sm-TSP2	2–24(6.8)	15.7±7.6	16.5	**21.4**	**0.306**	90671±49179	107768	**4.0**	**0.968**	4537±5752	3341	**41.5**	**0.326**
**Trial III (LDC)**													
Thi	2–11(2.7)	8.33±2.8	9	**-**	**-**	21176±16629	23966	**-**	**-**	637±643	560	**-**	**-**
Sj-TSP2	6–12(1.6)	8.30±2.1	7.5	**16.7**	**0.679**	20513±23301	10921	**54.4**	**0.326**	450±561	179.3	**68.9**	**0.251**
Sm-TSP2	3–10(1.5)	7.30±2.6	7.0	**22.2**	**0.266**	11726±13051	6962	**70.9**	**0.067**	378±356	340	**41.0**	**0.235**

Note: Thi, thioredoxin, as tag protein expressed in *E. coli* was used as a control protein. Sj-TSP2, Sj-TSP-2e; Sm-TSP2, Sm-TSP-2. Med, median; Redu, reduction = (1-number of median worms or eggs in experimental group/number of median worms or eggs in control group)×100%; ND, no data. HDC, high dose challenge with 35 cercariae; LDC, low dose challenge with 12 cercariae.

## Discussion

We report that vaccinating mice with the large extracellular loop (LEL) of Sj-TSP-2e (expressed in *E. coli*) from *S. japonicum* did not induce consistent protective efficacy. Yuan et al. [9] cloned and expressed Sj-TSP-2d and showed that the molecule induced significant levels of protection, although Sj-TSP-2c [8] and, as shown here also, Sj-TSP-2e did not induce any significant protection, suggesting that different subclasses of Sj-TSP-2 may stimulate different levels of protective efficacy. The serum IgG titres in the mice we vaccinated with Sj-TSP-2e were elevated after the second vaccination (Fig. 7) and ranged from 1∶320,000 to 1∶640,000 (data not shown) before challenge, which were lower than those in mice vaccinated with Sm-TSP-2 in the study by Tran et al. [5]. We used the same quantity of Sm-TSP-2 and the same vaccination procedure and schedule, and the serum titre in the group reached 1∶320,000 against Sm-TSP-2 (data not shown). We also expressed Sj-TSP-2e in yeast (Fig. 4, lane 5) and the resulting 6His tagged protein a stimulated a very IgG high titre (>1∶3,000,000; data not shown) of murine antibodies. However, the mice were not protected against a challenge infection with *S. japonicum* (data not shown), indicating the serum titres (ranging between 1∶320,000 to 1∶3,000,000) showed no correlation with protective efficacy.

We showed that Sj-TSP-2e specifically stimulated mice to produce IgG1 and IgG2 antibodies, which was similar to that reported with Sm-TSP-2 in vaccinated mice [5]. The levels of the two subclasses of IgG are recognised as markers of Th1 (IgG1) and Th2 (IgG2) responses [19], [20], which normally inhibit each other. It would be valuable to determine whether Sj-TSP-2e can actually stimulate the two types of T cell. Generally, a Th1 response is positively associated with protective immunity, whereas a Th2 response likely benefits parasite survival in the mammalian host [21], [22]. Based on previously published data and those presented here, we believe it is premature to speculate whether Sj-TSP-2 is suitable for vaccine development against schistosomiasis japonica. Given the protein is located in the schistosome tegument and appears to be highly polymorphic, it likely plays a role in avoidance of the host immune response. It is not known whether the protein is important for parasite growth and what its relationship is with other tetraspanins in schistosomes. A recent study using RNA interference to silence expression of *Sm-TSP-2* mRNAs in *S. mansoni* adults and schistosomula resulted in impaired turnover of the tegument apical membrane complex, suggesting Sm-TSP-2 plays an important structural role impacting on the development, maturation or stability of the schistosome tegument [6].

As most sequences of the Sj-TSP-2 subclasses are identical, it was difficult to design specific primers to distinguish each of the subclasses by real time PCR. Instead, we designed a pair of universal primers which amplify all the subclasses of Sj-TSP-2 to show the overall expression of the genes. The expression levels of the gene(s) were normalized against cercariae which showed that the gene expression in males was 5 times higher than in females (Fig. 3). However, EST sequencing of individual males and females showed that each of the subclasses was different in terms of their frequency (Table 2), indicating these subclasses of Sj-TSP-2 are differentially expressed in male and female *S. japonicum*. The results showed that Sj-TSP-2e was predominantly expressed in females, suggesting the gene is likely associated with female development. This can also help to explain the minimal amount of stain evident in sections of males, compared with female worms, in the immunohistological analysis undertaken with specific anti-Sj-TSP2e antibodies.

The polymorphism of Sj-TSP-2 is a major concern for developing this molecule as an anti-*S. japonicum* vaccine. We aligned 211 clones from *S. japonicum* and showed that there are at least 7 different clusters of Sj-TSP-2 cDNA sequences. It is not clear whether the variation is caused by transcription from different genes or alleles. BLAST analysis of *S. japonicum* genomic sequences with a full-length *Sj-TSP-2* cDNA including the 3′UTR (EF634060) found only one copy of the gene with 5 exons in the genome (data not shown), suggesting that the variation is likely due to the presence of different alleles. If so, it will be informative to determine how many alleles of Sj-TSP-2 exist. Combining our findings with those of Cai et al [8], there are a total of 9 different clusters/subclasses, suggesting at least 9 alleles based on the variable region of Sj-TSP-2. In addition, we identified one single worm that had three different cDNA clusters (data not shown), which is similar to previous findings [8] which showed that two *Sj-TSP-2* genomic fragments could be amplified from a single worm, indicating *S. japonicum* has 2 copies of the *Sj-TSP-2* gene at different loci in its genome, with one copy having two alleles and another having the same allele.

Considering all the sequences have the same number of nucleotides in the ORF encoding homologous protein sequences, these variable sequences have likely arisen from different alleles of *Sj-TSP-2*. To determine whether they belong to different genes, we searched their identical sequences in the Genbank databases and aligned their 3′ UTRs. We found a variable region with different numbers of nucleotides in the 3′ UTR, with the variation paralleling that in the large loop (Fig.S1); the gaps in some sequences indicate the possibility of the presence of different genes (Fig. S1). Two sequences - FN319825 and FN319885 - share the same ORF encoding Sj-TSP-2f, but have different 3′ UTR nucleotides, indicating the same protein is transcribed from different genes in the *S. japonicum* genome.

In contrast to Sj-TSP-2, there is evidence that Sm-TSP-2 exhibits very limited variation in its cDNA sequence suggesting that the gene in *S. mansoni* is conserved (Cupit PM et al. (2010) An investigation of polymorphism in the tetraspanin-2 gene of *Schistosoma mansoni* field isolates. Am J Trop Med Hyg Abstract Book, 59th Annual Meeting p123; Charles Cunningham, personal communication). This may reflect the fact that S. *mansoni* infects only humans and a limited number of other definitive host species, whereas *S. japonicum* utilizes a wide range of final hosts including humans and other mammals including cats, dogs, goats, horses, pigs, rats and water buffaloes. In China, *S. japonicum* can mature in over 40 species of domestic and wild mammalian hosts [23]. It is likely that the selection pressure provided by the various mammalian host species that *S. japonicum* infects promotes the development of mutated gene sequences as is evident in Sj-TSP-2, a feature that may be associated with its adaptation to the various host assemblages, a trait characteristic of a number of other parasitic helminths [24], [25], [26].

Signal prediction analysis showed the first 33 residues of the Sj-TSP-2 sequence represent a signal peptide which is encoded by the first exon of the genomic sequence. This region is highly conserved in both the Sj-TSP-2 and Sm-TSP-2 sequences (data not shown). The variable region is located in the middle of the TSP sequences and is encoded by three exons. The phylogenetic analysis of the large extracellular loop region we undertook showed that the 12 unique sequences occurring in *S. japonicum* fell into two poorly supported clusters, within which some subclustering occurred. Two clusters had clade credibility values over 95%: (Sj-TSP-2c, Sj-TSP-2h, Sj-TSP-2i) and (Sj-TSP-2g, Sj-TSP-2f, CAX70617_Sj), Fig. 2.

It is noteworthy that a number of different tetraspanins (at least 23 different genes deposited in the GenBank databases), including Sj23 and Sj25, have been described in *S. japonicum* [4], [5], [8], [9], [27], [28]; a recent report showed that Sj23 was able to induce very encouraging levels of protection in Chinese water buffaloes experimentally challenged with *S. japonicum* [27]. It is important to determine how many types of *Sj-TSP-2* gene are present in *S. japonicum* and the distribution of these different subclasses/alleles in *S. japonicum* populations from different endemic areas. This is fundamental for further consideration of Sj-TSP-2 as a vaccine target, and such analysis will increase knowledge of the possible role of this polymorphic protein in modulating immunity and the mechanisms whereby schistosomes are able to evade the host immune response.

## Supporting Information

Figure S1 Parallel alignment of variable and 3′ UTR sequences of 7 clusters of Sj-TSP-2. The comparison shows some gaps in some sequences indicating the possibility of the presence of different genes. GenBank accession numbers for the sequences are listed on the left hand of panel A.
(TIF)

Figure S2 Transmembrane domains of Sj-TSP-2e generated by MacVector 8.0.
(TIF)
