# Supplementary figures and images for: Inconsistent Protective Efficacy and Marked Polymorphism Limits the Value of Schistosoma japonicum Tetraspanin-2 as a Vaccine Target
Source: PLoS Negl Trop Dis. 2011 May 31;5(5):e1166. doi: 10.1371/journal.pntd.0001166 (PMC3104969; doi:10.1371/journal.pntd.0001166)

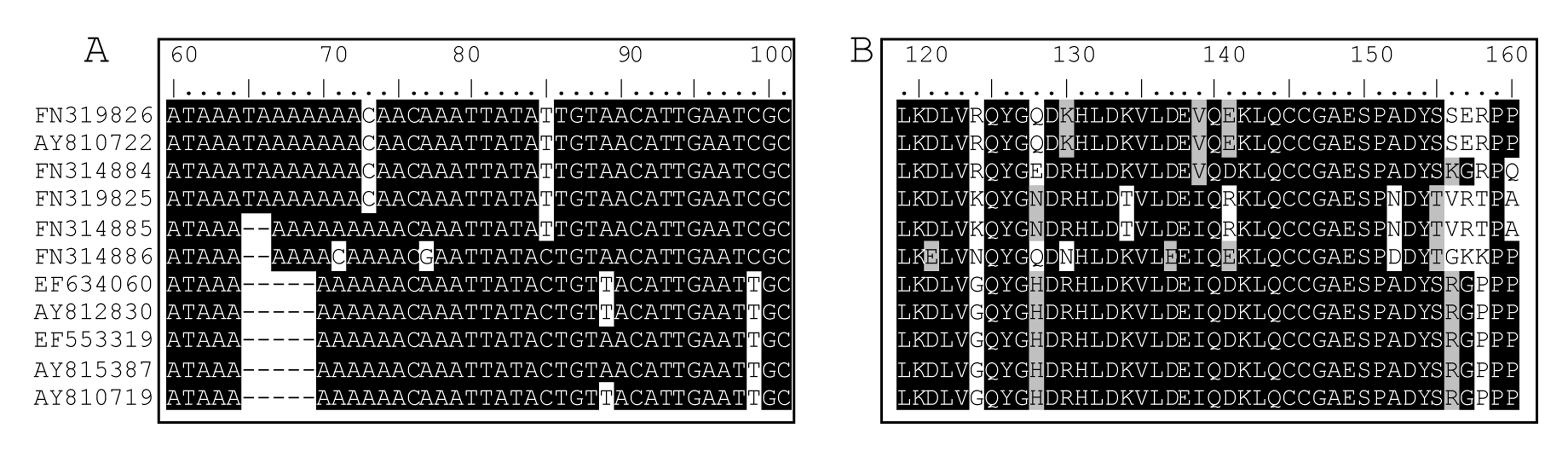

Supplement: Figure S1 — Parallel alignment of variable and 3′ UTR sequences of 7 clusters of Sj-TSP-2. The comparison shows some gaps in some sequences indicating the possibility of the presence of different genes. GenBank accession numbers for the sequences are listed on the left hand of panel A. (TIF) [file pntd.0001166.s001.tif]

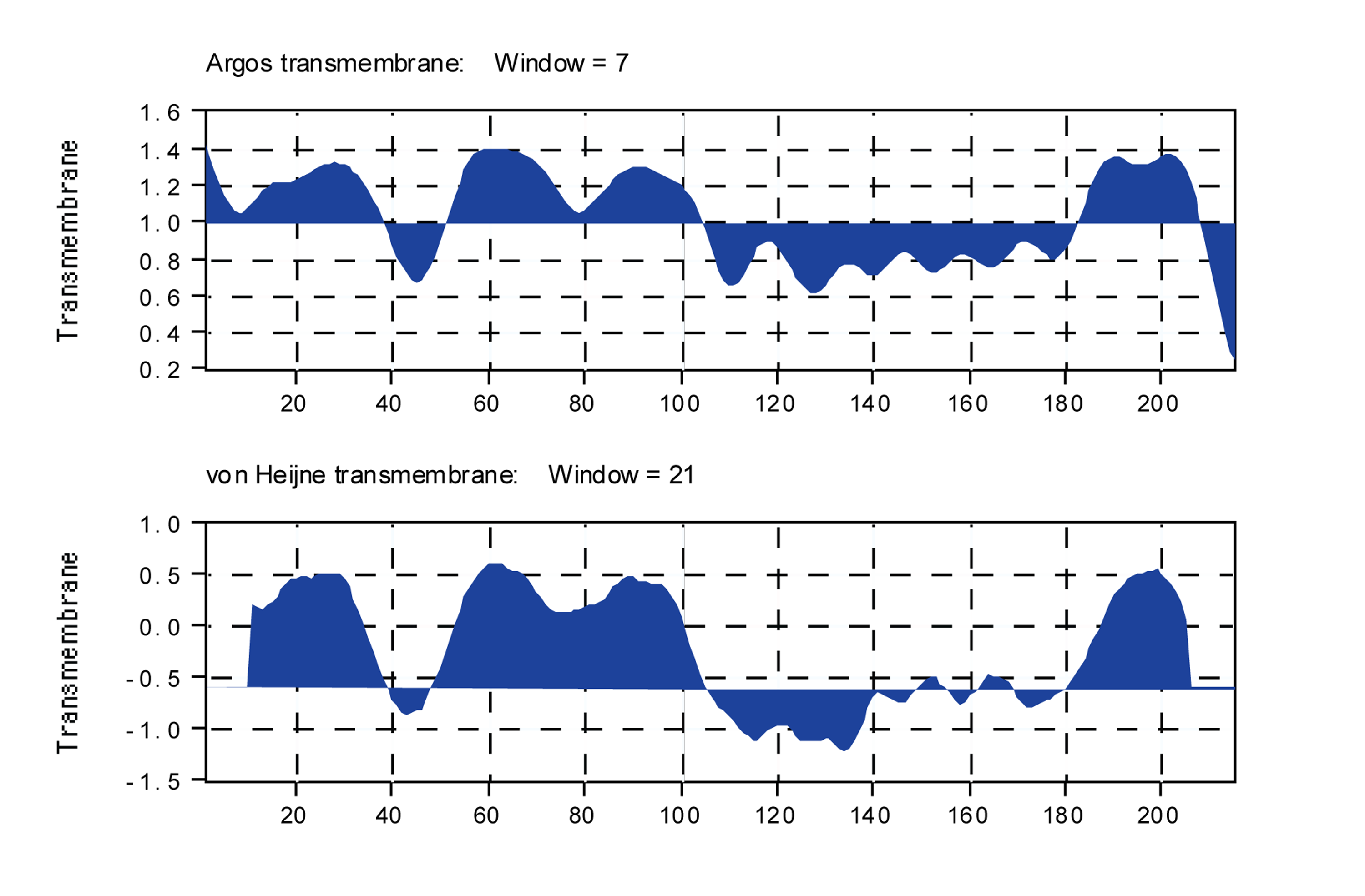

Supplement: Figure S2 — Transmembrane domains of Sj-TSP-2e generated by MacVector 8.0. (TIF) [file pntd.0001166.s002.tif]
